# Sensor-based characterization of construction and demolition waste at high occupancy densities using synthetic training data and deep learning

**DOI:** 10.1177/0734242X241231410

**Published:** 2024-02-22

**Authors:** Felix Kronenwett, Georg Maier, Norbert Leiss, Robin Gruna, Volker Thome, Thomas Längle

**Affiliations:** 1 Fraunhofer IOSB, Institute of Optronics, System Technologies and Image Exploitation, Karlsruhe, Germany; 2 Fraunhofer IBP, Institute for Building Physics, Holzkirchen, Germany

**Keywords:** Machine learning, object detection, synthetic data, sensor-based sorting, construction and demolition waste, circular economy

## Abstract

Sensor-based monitoring of construction and demolition waste (CDW) streams plays an important role in recycling (RC). Extracted knowledge about the composition of a material stream helps identifying RC paths, optimizing processing plants and form the basis for sorting. To enable economical use, it is necessary to ensure robust detection of individual objects even with high material throughput. Conventional algorithms struggle with resulting high occupancy densities and object overlap, making deep learning object detection methods more promising. In this study, different deep learning architectures for object detection (Region-based CNN/Region-based Convolutional Neural Network (Faster R-CNN), You only look once (YOLOv3), Single Shot MultiBox Detector (SSD)) are investigated with respect to their suitability for CDW characterization. A mixture of brick and sand-lime brick is considered as an exemplary waste stream. Particular attention is paid to detection performance with increasing occupancy density and particle overlap. A method for the generation of synthetic training images is presented, which avoids time-consuming manual labelling. By testing the models trained on synthetic data on real images, the success of the method is demonstrated. Requirements for synthetic training data composition, potential improvements and simplifications of different architecture approaches are discussed based on the characteristic of the detection task. In addition, the required inference time of the presented models is investigated to ensure their suitability for use under real-time conditions.

## Introduction

The construction sector is one of the most resource-intensive sectors of the economy. The stock of buildings and infrastructures in Germany, at around 28 billion tonnes (as of 2010, Umweltbundesamt (UBA)), is a significant man-made storage of raw materials that can be recycled when they are no longer needed (Wilke, 2013). Construction waste then accumulates as construction debris, road debris, soil and stones and as construction site waste. In 2018, mineral construction waste including excavated soil – that is, soils and stones – was the most important waste group in Germany in terms of volume, at 218.8 million tonnes. Among the mineral construction waste, 59.8 million tonnes were construction debris. Of this, 46.6 million tonnes (77.9%) could be recycled, and another 9.6 million tonnes (16.0%) were used, for example, in landfills or backfilled. The remaining 3.6 million tonnes (6.1%) were disposed of in landfills.

However, despite the high recycling (RC) rates, there are three major problems with the RC of construction waste. Firstly, the quality of RC materials often does not match that of primary raw materials. Hence, they are often used in low-value applications such as landfilling or backfilling. Secondly, the highly heterogeneous composition of construction waste prevents its high-value use. Thirdly, construction waste contains pollutants that preclude its use in many areas. For example, the majority of gypsum-based construction waste (over 50%) is currently disposed of in landfills (Wilke, 2013).

Due to the challenges described above, the characterization and sorting of construction waste components are of great importance for the reuse in high-value applications. To produce recycled construction materials with defined properties from the secondary raw material, the material waste stream requires processing. The quality of the recycled material depends on the input stream, especially whether it is homogeneous or not. Heterogeneous input material requires complex processing steps to achieve the desired requirements for the RC material. Therefore, the choice of technology for the processing of construction and demolition waste (CDW) depends on the characteristics of the raw material and the desired product quality (Müller and Martins, 2022).

Basic processing methods for CDW include crushing, screening (pre-sorting to limit maximum particle size) and further sorting, which essentially involves separating materials by type based on other physical characteristics. Examples of physical characteristics of CDW are its particle density and colour. In addition, the shape and exact size of the particles can also be taken into account (Müller and Martins, 2022). However, in addition to these mechanical sorting methods, sensor-based sorting is becoming increasingly important as it allows making sorting decisions based on further characteristics. The benefits of sensor-based sorting have been proven in other waste management scenarios. The process has the potential to provide solutions for achieving a true circular economy by sorting CDW into defined target fractions. To do this, the components contained must be reliably detected and characterized (Hoffmann Sampaio et al., 2021).

Sensor-based detection of a material flow serves not only as a basis for decision-making in sensor-based sorting but also as a general source of information for characterizing the CDW stream. Foreign and hazardous substances can be detected by the sensor. In addition, the information obtained allows statements to be made about the composition of a material stream. This information can be used for further processing steps or the selection of a suitable RC path.

### Current state of the art

Various imaging sensor principles are used to characterize the components of CDW streams. Among other things, the selection depends on the material flow and the respective characterization task. Hyperspectral cameras in the short-wave infrared and near-infrared range offer the possibility of material-specific differentiation (Serranti et al., 2015; Xiao et al., 2019). For many tasks, however, differentiation based on colour and shape characteristics is sufficient. For this purpose, colour cameras are a cost-effective option (Lin et al., 2022).

The goal of sensor-based CDW characterization is the detection of individual objects, for example, in the form of a rectangle enclosing the object (bounding box), as well as the correct classification, that is, the assignment of the object to a material class such as gypsum, brick, concrete or sand-lime brick. The information obtained can be used to monitor material flows and their composition and to implement sorting using suitable actuators.

**Figure 1.**
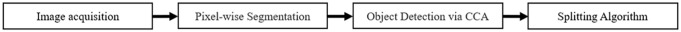
Classic image processing chain of a visual inspection system consisting of image acquisition, pixel-wise segmentation using colour features, identification of object boundary via CCA and detection of individual objects by means of splitting algorithms. CCA: connected component analysis.

However, in images with a high material throughput, the densely packed instances overlap, which means that classical image processing algorithms are increasingly reaching their performance limits regarding the differentiation of object instances. Such a conventional image processing pipeline for CDW characterization is shown in Figure 1. After image acquisition, segmentation is carried out in the form of a pixel-by-pixel classification based on simple colour features. For the detection of contiguous objects, a connected component analysis (CCA) is executed.

The CCA result reacts sensitively to overlapping objects, as object clusters are wrongly perceived as a single object due to a connection analysis. Additional splitting algorithms are therefore necessary for the further detection of individual object instances.

Data-driven deep learning approaches offer the potential to correctly differentiate complex, dense scenes of numerous overlapping objects. Due to the consideration of spatial information and correlations, object boundaries can be found more reliably. A disadvantage is the need for a large amount of training data from which correlations are learned during model training. To avoid manual labelling of the data, the idea of synthetically obtaining training data has been developed. Various approaches exist in this context, that is, the detection of overlapping, small objects and synthetic data generation.

In recent works, established deep learning architectures have already been used for object detection and instance segmentation of numerous small overlapping objects. Model training often relies on synthetic training data generation to avoid costly annotation. In (Toda et al., 2020), synthetic images were generated from individual grain seeds to train a Mask R-CNN for instance segmentation. The authors recommend focusing rather on a high variability of different object orientations and overlap examples than on a high number of different object textures when creating the training set. Synthetic data generation for images with homogeneous object clusters using single object instances is also discussed in (Wu et al., 2018). The data generation has an additional illumination transformation for realistic illumination and shadow properties of the training images.

### Contribution

In this study, various existing deep learning architectures for object detection were applied to the problem of CDW stream characterization to obtain information about their suitability. The basis of the model training was synthetically generated data, starting from individual, exposed object instances. The results allow drawing conclusions about suitable model architectures, possible task-specific improvements and the composition of the training data. The model performance was examined at different occupancy densities with the help of synthetic images. In addition, the real-time capability was investigated. To evaluate the training strategy based on synthetically generated training data, the transfer to real captured bulk material images was evaluated in terms of detection performance.

## Methods and materials

In the following, the selected architectures for object detection in the context of the characterization of CDW particles are presented. Subsequently, the procedure for synthetic data generation, used for model training, is explained.

### Suitable architectures for object detection

Deep learning architectures for object detection require a three-channel red, green and blue (RGB) image as input and deliver bounding boxes around detected objects as well as their class assignment combined with a confidence value as a result. Depending on the structure of the architecture, a distinction is made between one-stage and two-stage object detectors.

As the name already indicates, two-stage object detectors perform their prediction in two steps. In the first step, regions of interest (ROIs) are suggested. These ROIs are then evaluated in more detail and classified in a second step. A well-known representative that is considered in this study is the Faster R-CNN architecture (Ren et al., 2016).

One-stage object detectors perform the prediction of object positions and class membership in a single step. This makes them usually faster in their prediction, typically at the cost of worse performance in more complex detection problems.

Comparatively, two-stage architectures already filter out many regions through the region proposal, while one-stage architectures are confronted with all regions of the input image, potentially causing problems of class imbalance and multiple detections of individual objects. Due to the pre-filtering of the detection suggestions in two-stage architectures, the head can be larger in order to extract richer features used for classification and location. Furthermore, two-stage detectors regress the object location twice, which leads to better bounding boxes.

Well-known architectures that have been selected for investigation are YOLOv3 (Redmon and Farhadi, 2018) and SSD (Liu et al., 2016). They differ significantly in the backbone used for feature extraction. Although YOLOv3 uses the darknet backbone consisting of several residual layers, the SSD architecture consists of a VGG-16 network known from object classification. A tabular comparison of the implementation of the architectures used in the investigations can be found in Table 1.

**Table 1.** Comparison of used object detection architectures.

	Faster R-CNN	SSD	YOLOv3
Architecture	Two-stage	One-stage	One-stage
Backbone	RestNet-50	VGG-16	Darknet-53
Topology	Feature pyramid network	Pyramidal feature hierarchy	Feature pyramid network

When choosing hyperparameters for model training, the properties of a particle characterization, which differs significantly from known object detection tasks of large image datasets, are considered. Existing model architectures were designed for large image datasets consisting of many images of different object classes, object scales and object perspectives. Contrary, specific properties of the given task are:

- Many small, overlapping objects of the same class
- Few classes
- Homogeneous, constant lighting
- Constant camera perspective
- Similar scaling of the objects

The model output consists of numerous possible bounding box candidates. These must be filtered in an additional step and reduced to the bounding boxes in which there actually might be an object. Therefore, an important post-processing step in object detection for these architectures is non-maximum suppression, in which non-relevant detections with redundant information are filtered out of the numerous estimated bounding boxes.

### Synthetic data as the basis for training

The annotation of images of crushed CDW is time-consuming due to the large number of small objects. Annotating a single image with more than 100 small objects can already take much more than an hour. For data-driven methods like training a deep learning approach, a high number of annotated images is needed. Therefore, it is worthwhile to use synthetically generated data for model training, especially in such an application.

An iterative algorithm is developed for the generation of synthetic bulk material images. For this approach, images of real CDW objects are required. To acquire these images, a line-scan camera with 400 pixels and a conveyor belt for transporting the objects were used and series of images with different occupancy densities captured. Firstly, individual objects are cropped from images with a very low density of occupancy and without overlaps. This can be achieved algorithmically by a simple foreground–background segmentation. A single object is randomly selected from the object pool and placed on a background at randomly selected coordinates, described in more detail below. The desired image can be configured with the help of several parameters:

- Maximum occupancy density
- Minimum distance between two objects
- Maximum degree of object overlap
- Percentage class ratio

The algorithm must fulfil two goals. Firstly, a suitable coordinate for placing an object must be found. The coordinates are selected by drawing the *x* and *y* coordinates from a uniform distribution:

(1) $$\rho \left(x\right)=\frac{1}{b-a}\cdot 1_{\left[a,b\right]}\left(x\right)$$

Based on the defined parameters listed above, the coordinates are checked for suitability. In particular, the maximum overlap with objects that have already been set and the distance to existing objects must be considered.

By inserting individual objects into an already existing background, various effects can occur. Especially at the edges, unwanted effects in the form of hard edges appear, making the image look unrealistic. Alpha blending is used to avoid this. With the help of the existing alpha mask of the individual objects, the non-transparent colour *C* can be calculated from two overlapping objects (with colour *A* and *B*) using

(2) $$C=\frac{1}{\alpha_{C}}\left(\alpha_{C}A+\left(1+\alpha_{C}\right)\alpha_{B}B\right)$$

with the resulting transparent colour

(3) $$\alpha_{C}=\alpha_{A}+\left(1-\alpha_{A}\right)\alpha_{B}.$$

As a result, the edges of the objects are displayed somewhat softer, which leads to a more realistic overall image. In addition, it makes object delineation more difficult, which can help models to learn more complex situations.

To avoid model overfitting and artificially increase the training data, data augmentation methods are often used, such as flipping, rotating and scaling existing training images. Synthetic data generation can produce an infinite amount of training images. Therefore, ideas from the data augmentation field can be adapted and applied to the synthetic data generation process. The goal is to learn robust detection models. Two approaches are therefore pursued during data generation, both with the intention of learning more robust models with the help of using Perlin noise (Bae et al., 2018).

Although it is a design goal that there exists a constant background in sensor-based sorting, series of recordings and statistical evaluation of background recordings reveal fluctuations in brightness in our scenario. To make the training data more variable and to increase robustness, an uneven background is created using fractal noise, generated by overlaying several Perlin noise functions of different frequencies.

**Figure 2.**
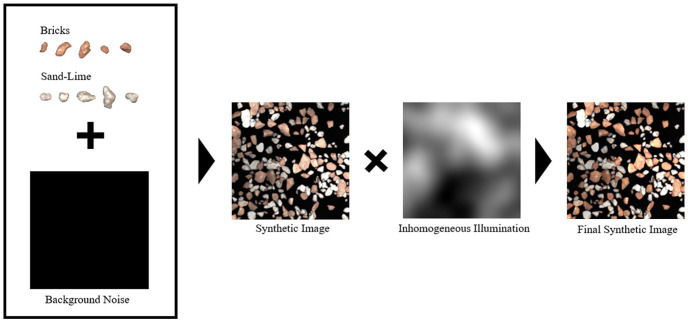
Schematic structure of the synthetic data generation including added noise and illumination effects.

In addition, an inhomogeneous illumination was simulated. The synthetically generated image is multiplied with a single low-frequency Perlin noise function. This causes a random but continuous change in brightness. The data generation process is shown in Figure 2.

**Figure 3.**
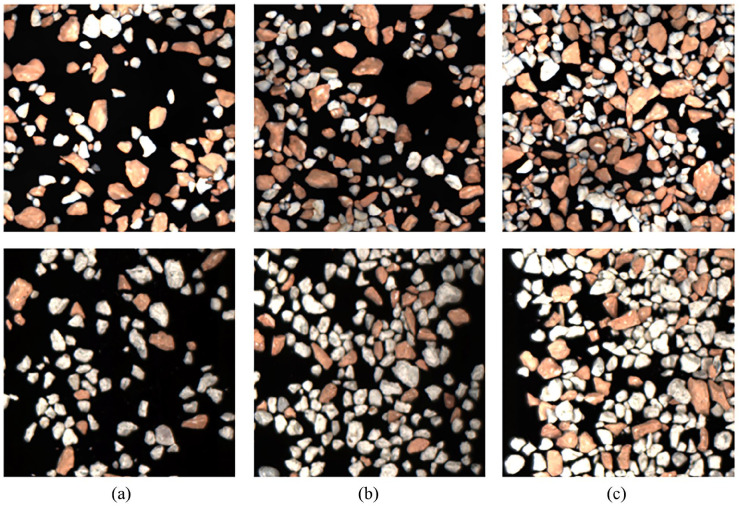
Top: synthetically generated images with brick and sand-lime brick with (a) 20, (b) 40 and (c) 60% occupancy density; bottom: real bulk images of brick and sand-lime brick with (a) 20, (b) 40 and (c) 60% occupancy density.

The generated images have a size of 300 × 300 pixels and contain brick and sand-lime objects in a mean ratio of approximate 50–50%. This prevents over-fitting to one class. A pixel-precise annotation of the images is automatically generated for the synthetical images during generation, which is required for the model training. Examples of generated images with different occupancy densities are shown in Figure 3. Overall, 5000 training, 500 validation and 500 test images were generated and used for the training and evaluation. Images with 20, 30, 40, 50 and 60% occupancy density (Δ density = ±5%) are generated. The evaluation is also done for these five occupancy densities.

For each architecture, different models were trained on the synthetic training dataset, including images with 20, 30, 40, 50 and 60% occupancy density. All three trained models are then evaluated on synthetic and real test images of the same occupancy density. In particular, the transfer of the models trained on synthetic data to real images provides information on the practicability of the training pipeline presented, consisting of synthetic data generation. In addition to the general suitability of the architectures for the existing detection task, conclusions can be drawn about the composition of the synthetic training dataset.

## Results

In the following, the results of the investigations are presented. Firstly, the models trained on synthetic data are evaluated and comparatively assessed. This is followed by an evaluation of the detection performance on real data. This allows to derive requirements for the generation of the synthetic training data and to make statements about the transfer capability of the learned features.

The mean average precision (mAP) is determined as a metric for detection performance. It is calculated from the area under the precision-recall curve. The averaged mAP over different intersection over unions (IoUs), which is often used in object detection, is not suitable for the observation and evaluation of a very high number of small objects, since false detections and grazing are not weighted sufficiently. Therefore, more strict metrics are used with an IoU equal to 75 and 90%. Objects are evaluated as correctly recognized (true positive) if the ground truth bounding box, that is, the ideal, minimum rectangle in which all pixels of the object lie, overlaps 75 or 90% with the estimated bounding box of the learned model.

### Evaluation on synthetic data of different occupancy densities

The strict metric mAP_IoU=0.9_ is used for evaluation on synthetic data. Due to the high number of small objects, differences in detection performance can thus be detected. It allows statements about the accuracy of the learned bounding boxes. Furthermore, different metrics were recorded to evaluate the recognition performance. This makes it possible to better detect objects that are not recognized at all.

The results in Table 2 show that all three architectures have good detection performance on the synthetic test images. Almost all objects are detected and correctly classified. This is supported by considering more tolerant metrics, for example, with a tolerated overlap of the estimated and real bounding box of 75%. There are only minor quantitative differences between the models. The architectures detect a similar number of object instances. Differences exist in the accuracy of the estimated bounding boxes, visible when considering the more restrictive metric. The Faster R-CNN architecture estimates the bounding boxes most accurately, followed by YOLOv3 and SSD.

**Table 2.** Achieved mAPs at different occupancy densities, each differentiated between synthetic and real test data and the three architectures: faster R-CNN, SSD and YOLOv3.

Faster R-CNN		Synthetic test data			Real test data		
		Occupancy density of the test dataset			Occupancy density of the test dataset		
		20%	40%	60%	20%	40%	60%
Occupancy density of the training dataset	20%	0.935	0.813	0.601	0.915	0.824	0.625
	40%	0.937	0.854	0.641			
	60%	0.924	0.841	0.654			
		mAP_IoU=0.9_			mAP_IoU=0.75_		
SSD		Occupancy density of the test dataset			Occupancy density of the test dataset		
		20%	40%	60%	20%	40%	60%
Occupancy density of the training dataset	20%	0.722	0.442	0.186	0.807	0.741	0.584
	40%	0.771	0.572	0.287			
	60%	0.707	0.543	0.352			
		mAP_IoU=0.9_			mAP_IoU=0.75_		
YOLOv3		Occupancy density of the test dataset			Occupancy density of the test dataset		
		20%	40%	60%	20%	40%	60%
Occupancy density of the training dataset	20%	0.734	0.493	0.209	0.842	0.767	0.592
	40%	0.812	0.646	0.332			
	60%	0.799	0.689	0.468			
		mAP_IoU=0.9_			mAP_IoU=0.75_		

mAP: mean average precision; IoU: intersection over union.

For the design of the training set, clear requirements can be formulated from the results. Models that are trained on images with a slightly higher occupancy density have a better detection performance on images with a slightly lower occupancy density. It is therefore recommended to select training data with 10% higher occupancy density than the expected occupancy density, as only then will the data contain sufficient situations with overlapping objects. However, choosing a too high occupancy density for training can also have a negative effect.

The additional consideration of the class-dependent metrics shows a similar detection performance of both material classes. In case of a possible overfitting to an overrepresented class, the training data would have to be adjusted or a more appropriate class weighting in the loss function would need to be used during training.

### Transfer to real data

The transfer of the model trained on synthetic data to real bulk material images is of particular interest. This way, the presented training strategy can be assessed regard to its use for real bulk solid detection problems. In addition to the comparison of the three architectures, statements can also be made about the synthetic data generated and an assessment of the annotation-free training strategy. For the investigation, one model each is trained on different occupancy densities (20–60%). This resulted as the most promising data set design from the results of the investigations on synthetic test data. The results and archived metrics of the investigations can also be seen in Table 2.

All models have in common that the accuracy of the estimated bounding boxes decreases when transferred to real data compared to the results obtained with synthetic data. One reason for this is overfitting on the individual object instances used for training data generation. For example, the bounding box around particularly long objects is estimated to be almost quadratic because the training data contains almost no elongated objects. Other reasons for the drop in the metric are architecture-dependent influences.

The Faster R-CNN model as a representative of the two-stage architectures has the best detection performance in comparison. The drop in the metric can be attributed to false additional detections of strongly overlapping sand-lime objects, in addition to the already known problem of the inaccurate bounding boxes.

For the SSD and YOLOv3 model, the described problem of false detections, especially for dense object regions, is even stronger. The YOLOv3 model seems to achieve good generalization and reliably detects the small objects on the real data. Problems exist in distinguishing between several objects of the same classes when there is a large overlap. In contrast, the SSD model has a poorer general performance in detecting the small objects, also noticeable when transferring to real data. The increase in these effects is the reason for the decline in metrics.

**Figure 4.**
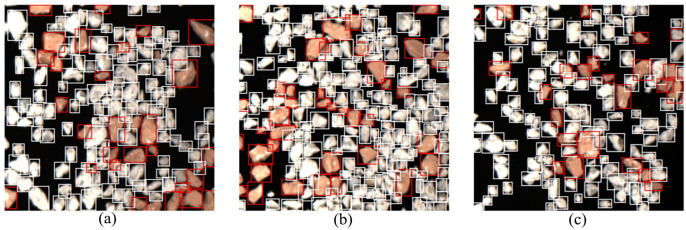
Comparison of example detections on real images of brick (red) and sand-lime (white) for the trained architectures; (a): faster R-CNN, (b): SSD and (c): YOLOv3.

Requirements for model selection can be derived from the results. The two-stage architecture has the best detection performance, since the transfer to real data also succeeds best due to a high generalization. One-stage architectures achieve a lower accuracy of the estimated bounding boxes and the detection of all objects during the transfer. Due to their lean architecture, they tend to overfit on the training data and fail to classify new unknown shapes and structures as objects (Figure 4).

### Model optimization

All models have a good detection performance, which motivates the use in the characterization of CDW. From the observations it can be concluded that also slimmer models might be sufficient for the discrimination and detection of CDW. Also motivated by the simplification of the current detection task compared to the existing large image data sets like ImageNet and COCO, an adaptation of the architectures is obvious. In this way, the smaller models can become faster and be used in a real-time capable system, for example, in a sensor-based sorting system.

A possible reduction concerns two areas. The backbone, which is responsible for feature extraction, can be slimmed down. Due to fewer classes to be expected as well as simpler object shapes, only a lower decline in the detection is to be expected. The second part concerns the head, which is responsible for the detection of the objects. In the example of YOLOv3, it has three outputs that cover the detection of three different object scales. In the existing data set, the objects have similar sizes, so different scales do not have to be covered. This motivates the simplification to only one output, which is specifically intended for the detection of small objects.

The above considerations were implemented with the YOLOv3 architecture. Layers were removed from the existing backbone, and the scaled output was removed and reduced to one output. The modified model architecture was then trained on the new data set of different occupancy densities. The weights to be trained were reduced from over 60 million to about 1.5 million. The new model was evaluated for its detection performance on the synthetic and real test images. The detection performance was similar to that of the unmodified model with a general small decrease in metrics due to more inaccurate bounding boxes. The comparison can be seen in Table 3.

**Table 3.** Comparison of detection performance between original and modified YOLOv3 architecture.

	Synthetic test data			Real test data		
	Occupancy density of the test dataset			Occupancy density of the test dataset		
	20%	40%	60%	20%	40%	60%
Original YOLOv3	0.782	0.662	0.479	0.842	0.767	0.592
Modified YOLOv3	0.775	0.617	0.438	0.797	0.665	0.524
	mAP_IoU=0.9_			mAP_IoU=0.75_		

mAP: mean average precision; IoU: intersection over union.

### Evaluation of real-time capability

Besides sufficient detection and classification accuracy, the real-time capability of the Deep Learning models under consideration plays an important role. When using the presented methods in a sensor-based sorting system, a result must be available between sensory detection and material ejection. Therefore, for the evaluation, a measurement of the inference time is performed in the optimized runtime environment TensorRT with a Nvidia GTX 3080 Ti graphics card. Images with a size of 300 × 300 pixels were used for the measurements.

The results show that the SSD and YOLO single-shot methods are significantly faster than the Faster R-CNN model. The YOLO model is slightly superior with an inference time of about 8.3 milliseconds, compared to the SSD with an inference time of about 9 milliseconds. However, the Faster R-CNN model is also capable of processing up to 79 FPS, reaching an inference time of 12.7 milliseconds. Particularly relevant in practice is the clearly recognizable trade-off between speed and accuracy, especially when comparing detection performance between one-stage and two-stage architectures.

**Figure 5.**
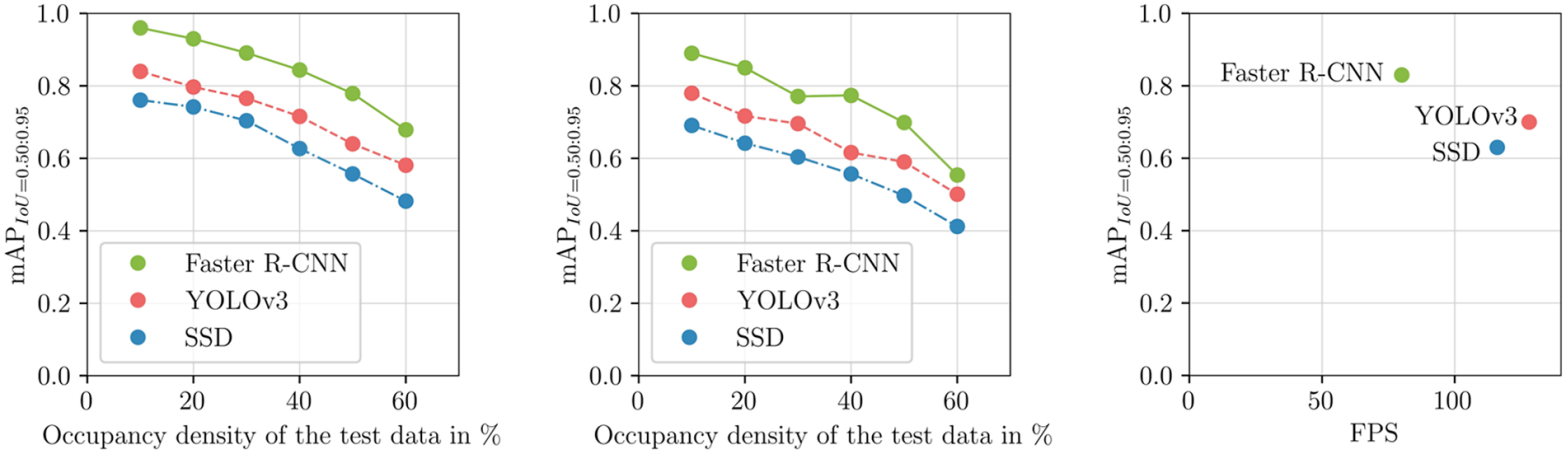
Left: comparison of archived mAPs of the best trained models at different occupation densities on synthetic and real construction rubble images. Visible decrease of detection performance by increased occupation density. Right: trade-off between speed and accuracy. mAP: mean average precision.

Whether the required real-time conditions are fulfilled depends not only on the choice of model and the hardware used but also strongly on the sensor used. Relevant key figures are the resolution and the readout speed. In addition, when using line-scanning sensors, as is typically the case in sensor-based sorting, it must be considered that lines need to be accumulated until the required two-dimensional input of a convolutional neural network is reached. This results in completely new considerations regarding the throughput achieved (Figure 5).

### Comparison with existing approaches and possible application scenarios

Existing detection pipelines and algorithms are reaching their limits, especially with high densities of CDW material streams. The results show the high potential of deep learning methods, especially in combination with the presented training strategy, consisting of synthetically generated training images. In addition, deep learning models have the theoretical ability to detect and distinguish CDW objects based on their shape and surface structure, regardless of their colour. This is a significant improvement over previous algorithms and is particularly important when inspecting other, more complex CDW material flows. The robustness in real application scenarios is demonstrated on real images that were acquired using a sensor-based sorting system. Nevertheless, an assessment of the change in detection performance with changes and variance in the input flow is yet to be performed. This offers potential for further investigations. The black box nature of the trained models makes it more difficult to rectify errors in the event of false detections, but the performance outweighs this potential disadvantage. Time-consuming manual annotation of training data can be avoided by the presented procedure.

The biggest identified remaining challenge is achieving low inference times. The architectures presented are computationally intensive. The inference time must be significantly reduced for use in a sensor-based sorting system. Possible strategies to overcome this challenge are streamlining the architectures or developing new approaches and models based on the specific requirements of bulk material detection.

## Conclusion and summary

The considered architectures are suitable for the characterization of CDW streams and can handle the specific requirements, for example, numerous overlapping objects of the same class. As expected, two-stage architectures have the best recognition performance and learn best a generalized representation of object shapes and classes compared to the single-stage architectures SSD and YOLOv3.

The demonstrated learning strategy with model training on synthetically generated data and subsequent transfer to real data showed high potential and minimizes the effort of manual data annotation. Special attention must be paid to a sufficient representation of the real object sizes and shapes. Additional variation through simulated inhomogeneous lighting effects increases the variability of the training data and leads to more robust models. The training data must cover a sufficient representation of different occupancy densities in order to recognize overlapping object scenarios sufficiently well. This specifically minimizes errors in the detection of overlapping objects of the same class, as well as general redundant detections in dense areas.

Investigation of the model runtimes further revealed the trade-off between detection performance and speed. As expected, one-stage architectures have a speed advantage over two-stage architectures with the problem of lower detection performance. The choice of architecture is therefore linked to the specific constraints of the task.

For use under real-time conditions while maintaining the required detection performance, adaptations must be made to the model architectures. Due to the special characteristics of the recognition task, such as few classes and similar object scales, even highly slimmed-down architectures might achieve sufficiently good recognition performance. A task-specific architecture implementation is therefore recommended. In addition to simplifying existing approaches, it is worthwhile to develop other representations and approaches for the detection of small CDW objects.
